# Attenuated sex-related DNA methylation differences in cancer highlight the magnitude bias mediating existing disparities

**DOI:** 10.1186/s13293-024-00682-4

**Published:** 2024-12-23

**Authors:** Jiaqi Zhou, Miao Li, Yu Chen, Shangzi Wang, Danke Wang, Chen Suo, Xingdong Chen

**Affiliations:** 1 State Key Laboratory of Genetic Engineering, School of Life Sciences, Human Phenome Institute, Fudan University, Shanghai, China; 2 Key Laboratory of Genetic Evolution & Animal Models, Kunming Institute of Zoology, Chinese Academy of Sciences, Kunming, Yunnan China; 3 Yunnan Key Laboratory of Animal Models and Human Disease Mechanisms, Kunming Institute of Zoology, Chinese Academy of Sciences, Kunming, Yunnan China; 4 Department of Psychiatry, Massachusetts General Hospital, Harvard Medical School, Boston, MA USA; 5 Stanley Center for Psychiatric Research, Broad Institute of MIT and Harvard, Cambridge, MA USA; 6 Analytic and Translational Genetics Unit, Massachusetts General Hospital, Boston, MA USA; 7 Fudan University Taizhou Institute of Health Sciences, Taizhou, China; 8 Department of Epidemiology, School of Public Health, Fudan University, Shanghai, China; 9 Shanghai Institute of Infectious Disease and Biosecurity, Shanghai, China; 10 Yiwu Research Institute of Fudan University, Yiwu, China; 11 National Clinical Research Center for Aging and Medicine, Huashan Hospital, Fudan University, Shanghai, China

**Keywords:** Sex differences, DNA methylation, Cancer, Gene expression, RNA-seq

## Abstract

**Background:**

DNA methylation (DNAm) influences both sex differences and cancer development, yet the mechanisms connecting these factors remain unclear.

**Methods:**

Utilizing data from The Cancer Genome Atlas, we conducted a comprehensive analysis of sex-related DNAm effects in nine non-reproductive cancers, compared to paired normal adjacent tissues (NATs), and validated the results using independent datasets. First, we assessed the extent of sex differential DNAm between cancers and NATs to explore how sex-related DNAm differences change in cancerous tissues. Next, we employed a multivariate adaptive shrinkage approach to model the covariance of cancer-related DNAm effects between sexes, aiming to elucidate how sex impacts aberrant DNAm patterns in cancers. Finally, we investigated correlations between the methylome and transcriptome to identify key signals driving sex-biased DNAm regulation in cancers.

**Results:**

Our analysis revealed a significant attenuation of sex differences in DNAm within cancerous tissues compared to baseline differences in normal tissues. We identified 3,452 CpGs (*P*_*bonf*_ < 0.05) associated with this reduction, with 72% of the linked genes involved in X chromosome inactivation. Through covariance analysis, we demonstrated that sex differences in cancer are predominantly driven by variations in the magnitude of shared DNAm signals, referred to as “amplification.” Based on these patterns, we classified cancers into female- and male-biased groups and identified key CpGs exhibiting sex-specific amplification. These CpGs were enriched in binding sites of critical transcription factors, including *P53, SOX2,* and *CTCF.* Integrative multi-omics analyses uncovered 48 CpG-gene-cancer trios for females and 380 for males, showing similar magnitude differences in DNAm and gene expression, pointing to a sex-specific regulatory role of DNAm in cancer risk. Notably, several genes regulated by these trios were previously identified as drug targets for cancers, highlighting their potential as sex-specific therapeutic targets.

**Conclusions:**

These findings advance our understanding of how sex, DNAm, and gene expression interact in cancer, offering insights into the development of sex-specific biomarkers and precision medicine.

**Supplementary Information:**

The online version contains supplementary material available at 10.1186/s13293-024-00682-4.

## Plain language summary

Sex disparities in non-reproductive cancers are well-documented across various aspects, including incidence, survival, mortality, and treatment outcomes. A deeper understanding of these differences could support the development of personalized therapeutic strategies. In this study, we conducted a comprehensive analysis of sex-related DNA methylation (DNAm) effects in nine non-reproductive cancers, comparing cancer tissues with paired normal adjacent tissues (NATs). Our findings revealed that DNAm differences between males and females were significantly reduced in cancerous tissues. The CpGs associated with this reduction were linked to pathways involving the tumor microenvironment. Additionally, we found that these sex differences in cancer were primarily driven by variations in the effect sizes of shared DNAm signals, allowing us to classify cancers into male-biased (BLCA, THCA, KIRP, LUAD, and HNSC) and female-biased (LUSC, LIHC, COAD, and KIRC) groups. Notably, we found that differential expression in cancers was correlated with differential methylation in a sex-specific manner, through concordant magnitude differences within each sex. Several of these genes, regulated by DNAm changes, were already targets of cancer drugs (e.g., *ECSCR, GATA2, and ERBB3*), highlighting the potential for developing sex-specific treatments. Overall, this research enhances our understanding of the role of DNAm in cancer and could contribute to more personalized therapies for both males and females.

**Supplementary Information:**

The online version contains supplementary material available at 10.1186/s13293-024-00682-4.

## Highlights

- The impact of sex on DNAm in cancers is pervasive and largely cancer-specific.
- DNAm-related sex differences were extensively attenuated in cancers.
- Amplification: magnitude differences in numerous shared DNAm signals dominate the sex effects on cancers.
- Correlated DNAm and gene expression pairs implicate concordant amplification effects.

**Supplementary Information:**

The online version contains supplementary material available at 10.1186/s13293-024-00682-4.

## Background

Sex disparities in non-reproductive cancers are well-recognized across various aspects, including incidence, survival, mortality, and treatment outcomes. For instance, a higher frequency of non-reproductive cancers occurs in males, leading to nearly twice the mortality rate compared to females [1–3]. Furthermore, females exhibit better overall survival outcomes from lung cancer surgery [4], while males benefit more from immunotherapies with immune checkpoint inhibitors in colorectal cancer [5]. Despite these notable sex differences in cancers, the underlying mechanisms of sex-specific effects on cancers remain largely uncharacterized. Understanding these molecular mechanisms could facilitate the development of sex-specific prevention and treatment strategies, thereby improving clinical care.

Previous studies have highlighted sex differences in the genomics of cancer tissues [5–10]. For instance, Yuan et al. [9] classified 13 cancer types into strong and weak sex-effect groups based on sex-biased molecular patterns, finding that 53% of clinically actionable genes exhibited sex-biased signatures. Similarly, Li et al. [10] identified a greater number of somatic single nucleotide variants in male patients than in females using a pan-cancer strategy. However, focusing solely on intra-tumoral comparisons may not fully capture the complexities of sex effects on cancers. Given the extensive reporting of sex-biased molecular signatures in human complex traits and tissues [11–14], it remains unclear whether such disparities are reorganized during cancer progression.

The impact of sex is shaped by a combination of individuals’ genomes, environmental effects, and their interplay. DNA methylation (DNAm), a stable epigenetic marker, can adapt genome function to changing environmental contexts. Previous studies have reported extensive sex differences in DNAm [15, 16]. Moreover, aberrant DNAm in cancer is recognized as a powerful target for clinical diagnostic, prognostic, and predictive biomarkers [17–20]. Thus, DNAm may serve as a promising endophenotype, connecting individuals’ genomes with sex-biased phenotypes in cancers. However, few studies have systematically examined sex differences in DNAm related to cancers.

In this study, we hypothesized that sex differences in DNAm levels are reorganized in cancers, resulting in ubiquitous sex-biased regulation of DNAm across cancers. To test this hypothesis, we conducted a comprehensive investigation of DNAm and its sex-specific effects in cancers using data from The Cancer Genome Atlas (TCGA). Our analysis included 3,435 cancer samples (N_female_ = 1,345; N_male_ = 2,090) and 482 paired normal adjacent samples (NATs) (N_female_ = 193; N_male_ = 289) from seven tissues across nine non-reproductive cancer types. The NATs served as controls in this study. To characterize the sex-specific role of DNAm in cancers, we aimed to answer two key questions: (1) How do the DNAm-related sex differences observed in cancers differ from those in normal tissues? (2) How do sex effects influence the aberrant DNAm observed in cancers?

## Methods

### Data collection

We obtained DNAm data and corresponding samples characteristics from the TCGA data portal (https://tcga-data.nci.nih.gov/tcga/). We collected nine major TCGA non-reproductive cancer types with sufficient sample sizes, comprising at least 10 samples each sex per diagnosis (cancer or NAT). In this study, we focused on the biological sex differences as defined by the sex chromosomes, XX (female) or XY (male). These cancer types include head and neck squamous cell carcinoma (HNSC), thyroid carcinoma (THCA), lung adenocarcinoma (LUAD), lung squamous cell carcinoma (LUSC), liver hepatocellular carcinoma (LIHC), kidney renal clear cell carcinoma (KIRC), kidney renal papillary cell carcinoma (KIRP), bladder urothelial carcinoma (BLCA), and colon adenocarcinoma (COAD). The DNAm profiles were generated using the Infinium Human Methylation 450K BeadChip. Each dataset was preprocessed separately and analyzed according to the workflow outlined below.

### Preprocessing for DNA methylation datasets

All analyses were performed in R (v.4.2.0). Raw IDAT formats were processed using ChAMP software (v.2.21.1) [21]. The probes were removed based on the following criteria: (1) probes with detection *p*-value greater than 0.01; (2) probes with less than three beads detected in at least 5% of samples per probe; (3) all non-CpG probes contained in our dataset; (4) single nucleotide polymorphism (SNP)-related probes [22]; (5) probes that map to multiple locations, according to Nordlund et al. [23]. The probes on the sex chromosomes were kept and further used to predict sex using the *estimateSex* [24] function from the wateRmelon R package [25]. Samples identified with sex chromosome aneuploidy or predicted sex different from their reported sex were removed. The beta-mixture quantile dilation (BMIQ) algorithm was applied to adjust the beta-values of type II probes into a statistical distribution characteristic of type I probes [26].

Batch effects introduced by different data sources were corrected using the *champ.runCombat* function. To capture potential underlying hidden confounding factors that may also influence DNAm, we performed smartSVA [27] on DNAm measurements. Putative surrogate variables (SVs) were estimated from DNAm values and parametrized to exclude DNAm variation linearly attributable to sex and diagnosis (cancer or NAT).

### Attenuation of sex differences in DNAm

To compare the extent of sex differences in DNAm between cancers and NATs, paired cancer and NAT subjects were extracted for use in this analysis. Separately in cancers and NATs, the number of sex-related differentially methylated positions (sDMPs) was calculated using the linear model in the limma R packages [28]. CpGs with a Bonferroni-corrected *P*-value (*P*_*bonf*_) less than 0.05 were considered significantly differentially methylated. The difference in the number of sDMPs for cancer vs. NAT subjects was calculated as the “true” difference.

To enhance the robustness of the results, a permutation-based approach (1000 times) was used to test the “true” difference. Each permutation randomly assigned “cancer” and “NAT” labels to subjects, keeping the number of subjects in each group consistent with the true number of cancer and NAT subjects. Permuted distributions of the difference in sDMPs between cancer and NAT subjects were generated for each cancer type. The two-tailed *P*-value were computed by testing the “true” difference against the permuted distribution. Significance threshold for altered sex-related DNAm among cancer and NAT samples was defined as a *P* < 0.05. If the permuted *P*-value was less than 0.05, with the number of sDMPs in cancers less than in NATs, it was considered significantly attenuated sex differences in cancers. Otherwise, a more widespread pattern of sex differences was represented in cancers.

The attenuated methylated positions (AMPs) refer to the sDMPs that exhibit the attenuation of sex differences between cancer and NAT subjects. To identify high-confidence AMPs, we extracted the sDMPs that were significantly methylated and had larger changes in NATs as compared to cancers. We calculated the occurrence of each “true” sDMPs identified in the control subjects within their respective permuted results. For each cancer type, those “true” sDMPs present in less than 95% of the 1000 permutations for NAT subjects were retained as AMPs. The AMPs were further subset into female-biased (higher methylation levels in females) and male-biased (higher methylation in males).

### Sex-stratified differential DNA methylation analyses

We performed differential methylation analysis using linear modeling approaches with the limma package in R [28] to examine the mean differences in DNAm levels (reported as Δβ) between cancer samples and NATs in females and males, separately. To ensure the robustness of our sex-stratified results, we also applied a conservative permutation test. We randomly shuttled “diagnosis” and “sex” labels within each cancer 1000 times and repeated the sex-stratified differential methylation analysis with the same linear model described above. Significance thresholds for sex-stratified differentially methylated positions (DMPs) were defined as both reaching the Bonferroni adjusted *P*-value and the permutation-based *P*-value less than 0.05. We defined hypermethylated DMPs as those with higher DNAm levels in cancers than in NATs, and hypomethylated ones as those with lower levels.

### Sex-by-cancer interaction analyses

Analyses for sex-by-cancer interaction DMPs were fit using the same model above, while adding the interaction term “sex*cancer” to tested the interaction effect between sex and cancer.

### Statistical analyses for the sample size bias issue

To address potential sample size bias between female and male subgroups that could impact the comparison of significant DMP numbers, we employed two stringent and complementary analytical approaches: (1) down-sampling analysis; (2) bootstrap-based approach.

For down-sampling analyses, the sex with larger sample sizes was randomly sampled to match the sex with smaller sample sizes within each type of cancer. This process was repeated 1000 times, and differential methylation analysis, as described in the previous section, was applied. Only THCA and LUAD had larger sample sizes in females, and down-sampling was applied to match the male sample sizes. For other cancers, male samples were randomly sampled using female sample sizes. The number of significant DMPs was calculated for each down-sampling iteration, and the distribution of DMP numbers was generated for each of the nine cancer types. A two-tailed *P*-value was obtained from testing the ‘true’ DMP number from the sex with smaller sample sizes against the down-sampled distribution, with the significance threshold defined as *P*-value less than 0.05.

However, the down-sampling approach was designed to generate the expected number of DMPs for the sex with larger samples when balancing the sample sizes bias. Importantly, this method may not be appropriate for assessing variance in expected numbers of DMPs between sexes. To address this concern, we also implemented a bootstrap-based approach. Subjects were sampled with replacement for each sex across cancers using the smaller sample sizes. After 1000 bootstraps, female and male distributions were compared with Wilcoxon rank sum tests to determine whether the number of DMPs differed by sexes (*P* < 0.05).

### Slope calculation for DNAm changes between sexes

Next, we compare the magnitude differences of DNAm changes (effect sizes) between females and males, utilizing sex-stratified differential methylation results through the following process. (1) Compute the regression slope of effect-size changes between female and male subgroups. The regression slopes and intercepts were calculated through orthogonal regression implemented using principal component analysis. An absolute slope > 1 indicates a larger effect in males, whereas an absolute slope < 1 indicates a larger effect in females. The 95% confidence intervals (CI) for these slopes were generated using the bootstrapped distribution (1000 times). (2) To evaluate distribution differences, we also compared the absolute effect size changes between sexes using Wilcoxon rank sum test. We corrected for multiple testing with the false discovery rate (FDR) procedure.

### Replication of sex-stratified differential DNAm

We obtained five additional DNAm datasets as the replicates from GEO database for LIHC (GSE54503) [29], LUAD (GSE66836) [30], COAD (GSE199057) [31], KIRC (GSE61441) [32], and THCA (GSE97466) [33]. Detailed information on these replicate datasets is provided in the Supplementary Table 7. The preprocessing steps were consistent with those applied to the discovery datasets. We evaluated the replication rate for sex-stratified DNAm results by quantified the π1 statistic in these five external datasets. Using the *P*-values derived from the replicate datasets, we calculated π1 using *qvalue* function from the qvalue R package (v.2.30.0) [34]. The π1 statistic represents the fraction of effects shared between the discovery and replicate datasets. Additionally, we calculated Spearman’s correlation for effect sizes of CpGs identified as DMPs in the discovery datasets between discovery and replication for females and males, separately.

### Amplification effects estimation

To examine the patterns of sex-specific DNAm effects across nine cancers, we utilized the data-driven covariance model proposed by Zhu et al. [35]. This model is based on the mash approach in the mashr R package (v.0.2.69) [36]. The mash algorithm allows for the quantification of effect-size heterogeneity and arbitrary patterns of correlation among sexes, thereby increasing power to detect both shared and sex-specific DNAm effects. We extracted effect sizes (DNAm changes) and corresponding standard errors from each sex across nine cancers to fit the covariance model. The mash model learns from the data by estimating mixture proportions of the predefined covariance matrices, organized by correlations ranging from −1 to 1 and relative magnitude of effects between sexes [35]. Using maximum likelihood, mash assigns posterior estimates for measurements, including posterior means, posterior standard errors, and local false sign rate (LSFR). It could reduce noise by shrinking effects towards zero and could reveal greater or lesser variation in effect sizes between sexes across cancers.

To determine the subset of CpGs for estimating sex-specific DNAm effects that better captures the distribution of weights between sexes across cancers, we examined two different subsets using nominal *P*-values of 1 (“all CpGs”) and 0.05 (“nominal CpGs”) as thresholds. These two subsets were randomly sampled using the non-redundant number of female- and male-related DMPs from sex-stratified results. We observed varying patterns of weights for null effect matrices across cancers (Supplementary Fig. 3a). The “all CpGs” subset captured more null effects, with an average weight of 25.23%, compared to “nominal CpGs”, which had only 0.20% on average. Additionally, most negatively correlated effects between sexes were more likely to be marginal effects that mostly cannot survival the multiple testing correction penalty (Supplementary Fig. 3b). It indicated that opposite directions of DNAm effects may not be a major driver of sex differences in cancers. Since the patterns of sex-specific DNAm effects were mostly consistent between these two subsets (Fig. 5c and Supplementary Fig. 3c), together with the aim of capturing more significant associations in cancers for both sexes and reflecting more causal signals for sex-specific DNAm effects, we decided to use the results of “nominal CpGs” for downstream analyses.

### Sex-heterogeneity of cancer-related DNAm changes

To evaluate sex-heterogeneity of cancer-related DNAm changes, we calculated the z-score and its associated p-value statistic based on the effect sizes (Δβ) and standard errors (SE) from sex-stratified results with Eq. (1).

1 $$z - score\, = \,\frac{{{\Delta }\beta_{female} - {\Delta }\beta_{male} }}{{\sqrt {SE_{female}^{2} + SE_{male}^{2} } }}$$

### Prioritize DNAm signals associated with cancer-related sex amplification effects

To effectively capture DMPs that prioritize robust sex amplification effects on DNAm in cancers, we integrated the results from sex-stratified analyses, the mash model, and sex-heterogeneity analyses.

We compared results from the “limma” and “mash” approaches and found that the “mash” method can overcome the statistical power restriction imposed by per-cancer available sample sizes (Spearman’s correlation between sample sizes and the number of sex-stratified DMPs, ρ_mash_ = 0.05 < ρ_limma_ = 0.42). Additionally, we observed a significant correlation between the number of sex-stratified DMPs identified in both analyses (Spearman’s ρ = 0.67, *P* = 2.83e-03). All sex-stratified DMPs identified by "limma" reached the significant threshold in the "mash" results (LSFR < 0.05).

We classified the identified sex-stratified DMPs into four main types (Supplementary Fig. 4): (1) sex-shared effects; (2) female-amplifiers; (3) male-amplifiers; (4) sex-opposite effects. The detailed criteria are as follow:

1. Sex-shared effects:
2. Significant in both sexes based on sex-stratified results
3. LSFR < 0.05 based on mash results
4. Sex-heterogeneity associated *P* ≥ 0.05
5. Consistent direction of DNAm changes between sexes
6. Female-amplifiers:
7. Significant in females based on female-stratified results
8. LSFR < 0.05 based on mash results
9. Sex-heterogeneity associated *P* < 0.05
10. The absolute DNAm changes were greater in females relative to males
11. Consistent direction of DNAm changes between sexes
12. Male-amplifiers:
13. Significant in males based on male-stratified results
14. LSFR < 0.05 based on mash results
15. Sex-heterogeneity associated *P* < 0.05
16. The absolute DNAm changes were greater in males relative to females
17. Consistent direction of DNAm changes between sexes
18. Sex-opposite effects:
19. Significant in both sexes based on sex-stratified results
20. LSFR < 0.05 based on mash results
21. Inconsistent direction of DNAm changes between sexes

### Cox proportional hazards regression

To evaluate the association of identified sex amplifiers with overall patient survival and recurrence, we applied Cox proportional hazards models to test the impact of DNAm underlying sex amplification effects on overall survival for each sex separately. We used Lasso regression for variable selection based on the *cv.glmnet* function in the glmnet R package, followed by multivariate Cox analyses using the *coxph* function in the survival R package. The *cox.zph* function was used to check the proportional hazards assumption for a Cox regression model fit.

### Preprocessing and statistical analyses for RNA-seq data

We obtained RNA-seq data (count data) and sample characteristics for nine included cancer types from the TCGA data portal (https://tcga-data.nci.nih.gov/tcga/). For each cancer type, log2-transformed counts per million (log2(CPM)) were obtained from read counts for gene filtering purpose using the *voom* function [37] in the limma R package. Genes with a CPM ≥ 0.1 in at least 30% of samples were retained, and those derived from mitochondrial DNA were removed. Sample connectivity (z-score) was calculated using the *fundamentalNetworkConcepts* function in the WGCNA R package [38], and samples with a z-score lower than -2 were excluded. Quantile normalization was then used to equalize distributions across samples. Additionally, the expression of the *XIST* gene (a female-specific gene) was assessed to contribute to sample identity verification. Batch effects were corrected using the *ComBat* function in the sva R package [39]. The putative hidden factors were estimated by smartSVA [27] on expression measurements and further regressed using a linear regression model.

For differential expression analysis, we performed a nonparametric method, the Wilcoxon rank-sum test, as recommended by Li et al. [40]. This method could achieve comparable and better power in detecting truly differentially expressed genes (DEGs) than parametric method under this phenomenon. Significant DEGs were determined with a Bonferroni-adjusted P-value less than 0.05.

### eQTM mapping

The association of DNAm with proximal gene expression was defined within ± 10 kb windows centered on the CpG locus. We obtained regressed DNAm and gene expression data from matched samples for eQTM mapping. Spearman’s correlation test was used to assess the correlation between DNAm and gene expression for the female and male subgroups, separately. Significant eQTMs were determined with a Bonferroni-adjusted P-value less than 0.05.

### X chromosome signatures

To assess the putative X chromosome inactivation (XCI) status for our sex-related DNAm effects, we obtained previously annotated XCI status across human tissues [41]. The known XCI categories, including 631 genes defined as escape (n = 99), variable escape (n = 101), or inactive (n = 431). We assessed enrichment of these sex-related DNAm effects for XCI status using Fisher’s exact test. The significance threshold was defined as a Bonferroni-corrected *P*-value less than 0.05.

The X chromosome is divided into five evolutionary strata, S1 to S5, arranged in order from the distal end of the long arm to that of the short arm [42–44]. Among these, S1 is the oldest stratum while S5 is the most recent one. Genes in older strata, known as the X conserved region (XCR), tend to get inactivated, while genes on more recent strata, known as the X added region (XAR), tend to escape inactivation. To investigate whether enrichment of sex-related DNAm effects on the X chromosome differs across evolutionary strata, we performed enrichment analysis using Fisher’s exact test. The significance threshold was defined as an FDR less than 0.05.

### Genomic annotation and transcription factor binding sites enrichment

Gene regulatory element annotations for candidate cis-regulatory elements (cCREs) were derived from ENCODE cCREs v3 [45] and the coordinates were converted to hg19 using UCSC’s liftOver tool. To annotate DMPs for cCREs, we extended the span of their genomic location by ± 200 bp and assessed enrichment with element regions using the LOLA R package [46].

To test the enrichment of transcription factor binding sites (TFBS) in sex-related DNAm effects (within ± 200 bp), we obtained the human cistrome regions from Vorontsov et al. [47], which provide the genome-wide maps of regions bound by TFs. We only used the cistrome regions supported by at least two experiments and at least two peak callers, ensuring the highest reliability, experimental, and technical reproducibility. In total, we obtained 137 TFs and tested enrichment using the LOLA R package [46]. We also performed Hypergeometric Optimization of Motif Enrichment (HOMER) (v 5.1) [48], to identify enriched conserved motifs within these X-linked sex-amplifiers.

Significant cCREs and TFs enrichment was defined as FDR less than 0.05.

### Functional enrichment

At the CpG level, functional enrichment of Gene Ontology (GO) biological processes was performed using R package missMethyl [49], which can correct the selection bias introduced by the different number of probes per gene. At gene level, we performed gene set enrichment analysis (GSEA) with the function *enricher* implemented in the R package clusterProfiler [50]. We obtained the hallmark gene sets and canonical pathways from MSigDB [51] and GO terms from GeneSetDB [52]. We defined significant threshold for GO enrichment was *P*-value less than 0.05. The druggable targets were obtained from CIViC database [53], which contained 513 genes.

## Results

### Data aggregation and study design

To investigate the sex effects on DNAm regulation in cancers, we obtained DNAm data and RNA-seq data with sufficient sample sizes (≥ 10 each sex per cancer) in nine major non-reproductive cancer types from TCGA (Supplementary Table 1). These cancer types include HNSC, THCA, LUAD, LUSC, LIHC, KIRC, KIRP, BLCA, and COAD.

**Fig. 1:**
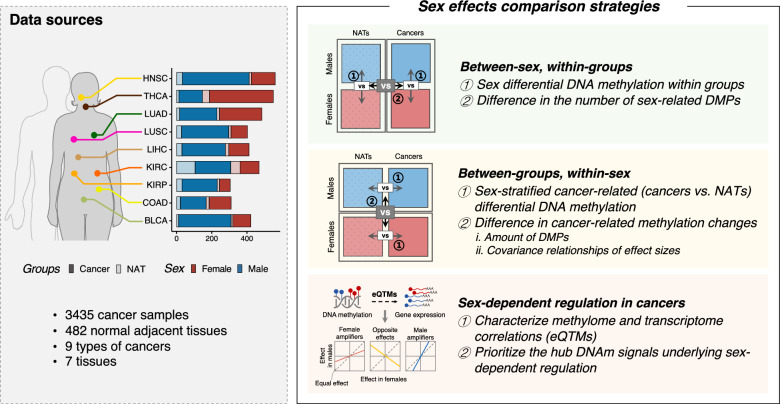
Study design. Schematic illustration of the key analyses used to investigate the impact of sex on DNAm in nine types of non-reproductive cancers. DNAm, DNA methylation; DMPs, differentially methylated positions

The overall study design is summarized in Fig. 1. First, we systematically compared the extent of sex differential DNAm between the paired cancers and NATs to determine whether the observed sex differences are reorganized in cancers. Next, employing a sex-stratified case–control strategy, we examined the amount, correlation, and magnitude of cancer-related DNAm changes between sexes to decipher how sex effects influence the aberrant DNAm patterns in cancers. Finally, we characterized correlations between the methylome and transcriptome, manifesting as eQTMs, to prioritize hub signals underlying sex-biased DNAm regulation in cancers. Through this DNAm-focused integrative analysis, our findings enhance understanding of molecular regulation in sex effects on cancers.

### Attenuation of sex differences in DNAm across cancers

**Fig. 2:**
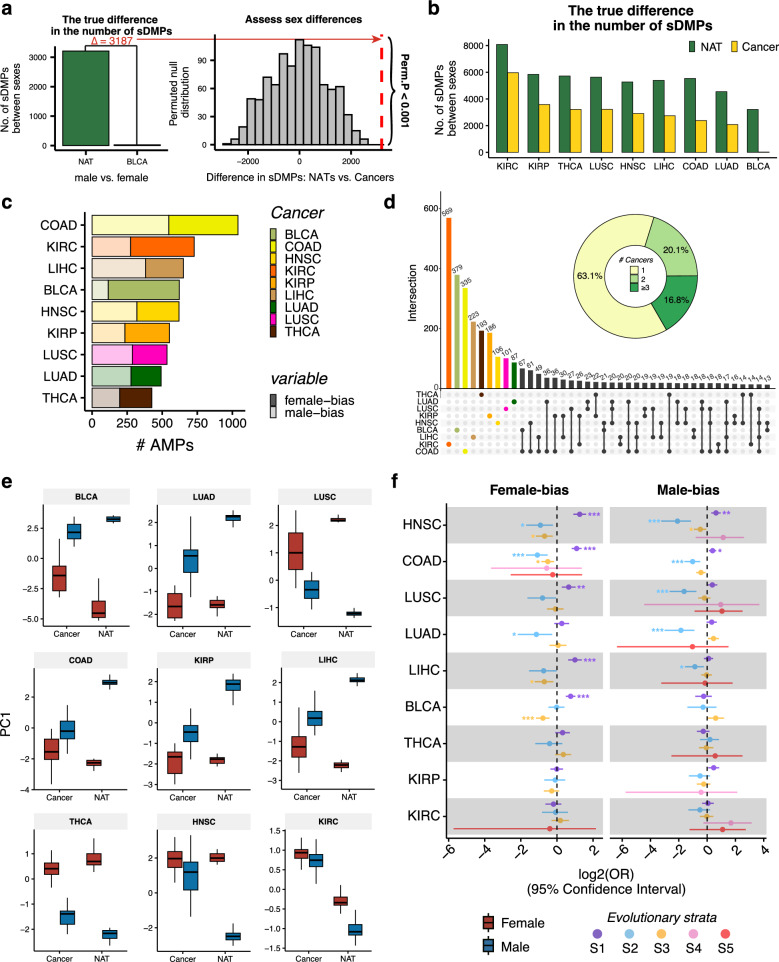
Attenuation of sex differences in DNAm across nine cancers. **a** Overview of methods for identifying statistically significant differences in the number of sDMPs across nine cancers. The comparison of BLCA versus NAT is used here as an example. Left, the true difference in the number of sDMPs between BLCA and NAT samples. Right, a permuted null distribution (1000 times) is then used to determine the significance of the difference in the number of sDMPs between BLCA and NAT samples. **b** The true differences in the number of sDMPs between the paired cancers and NAT samples across all nine cancers. All pairs reaching a permutation *P* < 0.001. **c** The number of AMPs identified in each cancer. The number of female- (higher DNAm levels in females) and male-biased (higher DNAm levels in males) AMPs are indicated. **d** UpSet plot of AMPs’ overlap between pairs of cancers. Dark dots and lines indicate that the set participates in the intersection. The doughnut chart indicates the cancer sharing profile of AMPs. **e**, PC1 of DNAm levels of AMPs between cancer and NAT samples across all pairs. **f** The enrichment of X-linked AMPs across these five evolutionary strata, labeled as S1 (the most conserved region) to S5 (the most recent region), arranged in order from the distal end of the long arm to the short arm. *FDR < 0.05; **FDR < 0.01; ***FDR < 0.001. *sDMPs* sex-related differentially methylated positions, *NAT* normal adjacent tissues, *AMPs* attenuated methylated positions, *PC1* principal component 1

To determine whether sex-related differential DNAm is reorganized in cancers, we compared the number of sex-related differentially methylated positions (sDMPs) between the paired cancer and NAT samples across nine cancer types, using a conservative permutation-based method (Methods). First, we quantified sex bias effects for cancers and NATs separately. In NATs, we identified 8,833 non-redundant sDMPs, ranging from 3,211 in BLCA to 8,084 in KIRC (Bonferroni-adjusted *P*-value, *P*_*bonf*_ < 0.05, Supplementary Table 2). Meanwhile, 6,361 sDMPs were identified in cancers, ranging from 24 in BLCA to 5,968 in KIRC (*P*_*bonf*_ < 0.05, Supplementary Table 2). Then, by comparing the number of sDMPs, we observed a significant reduction in the number of sDMPs in cancers compared with NATs for all nine cancer types (all permute *P*-value < 0.001, Fig. 2a, b, Supplementary Fig. 1a and Methods). The most pronounced reduction of sex differences in DNAm was observed in BLCA, with the fewest sDMPs identified in the cancer state (Fig. 2a). These results demonstrate a marked attenuation of sex differences at DNAm levels in cancers, yielding a more epigenetically homogeneous pattern during cancer progression.

To explore how sex-related DNAm changes were reduced in cancer, we employed a conservative filtering process to extract sDMPs exhibiting attenuation (Methods). To elucidate this procedure, we defined attenuated methylated positions (AMPs) as sDMPs that were significantly methylated in NATs but not in cancers. We identified 3,452 significant AMPs (*P*_*bonf*_ < 0.05), ranging from 426 to 1,040 AMPs per cancer (Fig. 2c and Supplementary Table 3). Regarding the direction of DNAm changes, 1,515 AMPs displayed higher methylation in males than in females and were classified as male-biased, while 1,970 AMPs showed higher methylation in females and were classified as female-biased. Overall, 63.12% (2,179/3,452) of these AMPs were cancer-specific (Fig. 2d and Supplementary Table 3). These AMPs exhibited greater DNAm changes in NATs than in cancers, representing the pattern of attenuation (Fig. 2e).

We found that 88.76% (3,064/3,452) of the AMPs were X-linked, a proportion that was not significantly different from the proportion of X-linked sDMPs in NATs (90.89%, 8028/8833; Fisher’s exact test, *P* = 0.23). Notably, 72% (442/614) of AMPs annotated genes were previously identified as X chromosome inactivation (XCI) genes, particularly the XCI inactive genes (Supplementary Fig. 1b, Supplementary Table 3 and 4). Positional enrichment on the X chromosome revealed that female-biased AMPs were strongly enriched in the X conserved region of the long arm in five out of nine cancers (HNSC, COAD, LIHC, BLCA, and LUSC), suggesting escape from XCI in cancers (false discovery rate (FDR)-corrected *P* < 0.05, Fig. 2f and Supplementary Table 4). Additionally, in contrast to male-biased AMPs, the detected female-biased AMPs predominantly resided in promoter and proximal enhancer regions, except for KIRC (FDR < 0.05, Supplementary Fig. 1c and Supplementary Table 4).

To investigate the biological processes contributing to this broad attenuation of sex differences in cancers, we performed functional enrichment analysis on the identified AMPs. Except for KIRC and THCA, we observed pervasive dysregulation of the negative regulation of transcription in the remaining seven types of cancers (Supplementary Fig. 1d and Supplementary Table 5). Notably, dysregulation of synapse-related pathways was identified in LUAD, LUSC, BLCA, COAD, and KIRP (Supplementary Fig. 1d and Supplementary Table 5). Emerging evidence indicates that crosstalk between the nervous system and cancer is a crucial regulator of the tumor microenvironment [54, 55]. These findings suggest that the sex differences in DNAm are reorganized in cancers, further contributing to the construction of the tumor microenvironment.

### Sex-stratified differential DNAm was largely cancer-specific

**Fig. 3:**
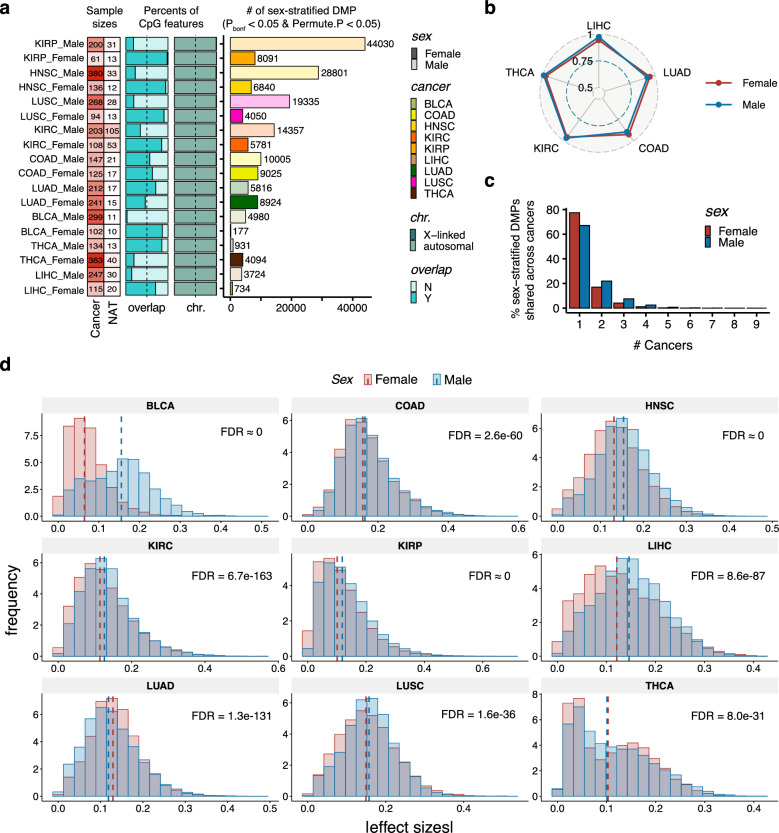
Sex-stratified cancer-related differential DNAm. **a** Discovery of sex-stratified DMPs across nine cancer types. Left, the number of sample sizes per sex and group (heatmap). Right, the number of identified fDMPs and mDMPs (Bonferroni-adjust *P* < 0.05 and permutation *P* < 0.05, histogram). Proportions of X-linked and autosomal f(m)DMPs (chr.) and of the overlap between fDMPs and mDMPs per cancer are indicated (stacked bar plots). **b** Replication rates (π1) for identified fDMPs and mDMPs in five types of cancers (LIHC, LUAD, COAD, KIRC, and THCA).** c** Cancer sharing profile of sex-stratified DMPs. **d** Absolute values of effect sizes for fDMPs and mDMPs. The dashed lines represent the median of effect sizes per sex. The FDR-corrected* P*-values according to paired Wilcoxon signed-rank tests are presented. *fDMPs* female-related DMPs, *mDMPs* male-related DMPs

To understand whether the alteration of DNAm in cancers involving sex-dependent regulation, a sex-stratified approach was applied to assess the differential methylation between cancers and NATs. We discovered a total of 36,693 female-related DMPs (fDMPs, *P*_*bonf*_ < 0.05 and permutation-based *P* < 0.05), with 177–9,025 fDMPs identified per cancer (Fig. 3a and Supplementary Table 6). Meanwhile, there were 88,811 male-related DMPs (mDMPs) identified (*P*_*bonf*_ < 0.05 and permutation-based *P* < 0.05), ranging from 931 to 44,030 mDMPs per cancer (Fig. 3a and Supplementary Table 6). Of these, essentially all (~ 99%) of the sex-stratified DMPs were located at autosomes (Fig. 3a). We quantified the replication rates for these sex-stratified DMPs in five external datasets for LIHC, LUAD, COAD, KIRC, and THCA and observed high replication rates (average π1 in females: 0.89; in males: 0.90; all spearman’s ρ > 0.70; Fig. 3b, Supplementary Fig. 2a and Supplementary Table 7).

Although the number of DMPs differed between males and females across cancers, an average of 23% of sex-stratified DMPs overlapped (Fig. 3a). The highest overlap was in COAD (43%), while BLCA had the lowest (3%; Fig. 3a). Furthermore, sex-stratified DMPs showed a pronounced cancer-specific pattern, with 77.41% of fDMPs and 67.07% of mDMPs being unique to individual cancers (Fig. 3c, Supplementary Fig. 2b).

The number of sex-stratified DMPs across cancers varies considerably, which may reflect sample sizes differences (Spearman’s ρ = 0.42, Fig. 3a). To ensure such sex bias were not driven by sample size differences, we performed 1000 rounds of down-sampling analyses and bootstrapping tests. Three types of cancers (COAD, LUAD, BLCA) exhibited significant sex differences in the number of DMPs based on the down-sampling analyses, with LUAD and COAD having more DMPs in females and BLCA having more DMPs in males (Supplementary Fig. 2c). Additionally, six other cancer types showed significant sex bias using the bootstrap-based approach (Supplementary Fig. 2d).

We next investigated whether the correlation and magnitude of DNAm changes differed by sex in cancers. The effect sizes were highly correlated between sexes across cancers, with ~ 99% of the sex-stratified DMPs showing consistent direction of DNAm changes (all Spearman’s ρ > 0.8, Supplementary Fig. 2e). Notably, the distribution of DNAm changes was significantly sex-biased across all nine cancers (paired Wilcoxon signed-rank test with FDR-corrected* P* < 0.05, Fig. 3d). The most prominent sex difference was observed in BLCA, with a significantly male-biased magnitude of DNAm changes (Fig. 3d). These results indicate largely consistent directional DNAm changes between sexes, but with varying magnitudes across cancers.

### Sex-by-cancer interaction effects on DNAm were ubiquitous across cancers

**Fig. 4:**
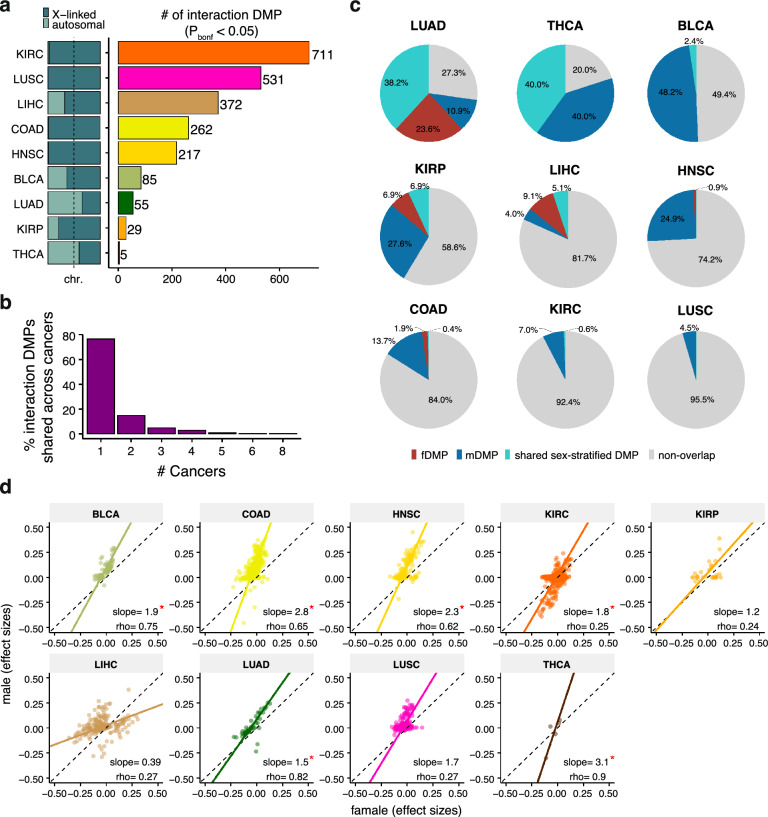
Sex-by-cancer interaction effects on DNAm. **a** Discovery of sex-by-groups interaction DMPs across nine cancer types. The proportions of X-linked and autosomal f(m)DMPs (chr.) per cancer are indicated (left, stacked bar plots). The number of interaction DMPs per cancer are shown (right, histogram). Legend colors are as in Fig. 3. **b** Cancer sharing profile of interaction DMPs. **c** Pie plots indicate the proportions of overlap between interaction DMPs and sex-stratified DMPs per cancer. **d** Cancer-related differential methylation effect size of females compared with males for the interaction DMPs per cancer. The slope (*S*) is calculated using principal components regression, with *FDR < 0.05 indicating that *S* is significantly different between sexes (see details in Methods). The dashed line represents equal effects between females and males when *S* = 1

We next evaluated the interaction effects between sex and cancer (cancer or NAT) across all nine cancers. There were 1,642 interaction DMPs identified, with 386 (23.51%) showing differential methylation in more than two different cancer types, suggesting cancer-dependent regulation (Fig. 4a, b and Supplementary Table 8). These interaction DMPs were largely X-linked (86.67%, Fig. 4a). Only a limited fraction of interaction DMPs overlapped with the identified sex-stratified DMPs, representing 15.11% for fDMPs and 30.49% for mDMPs on average (Fig. 4c). These suggest that these two analytical strategies complement each other and coordinately reflect the sex effects on DNAm in cancers.

We conducted functional enrichment analyses of the interaction DMPs across cancers, identifying significant enrichment in pathways associated with cell metabolism, cell cycle control, immune response, and tumor microenvironment interactions (Supplementary Table 8). Notably, synapse-related pathways were commonly dysregulated across most cancers, except THCA, underscoring the potential impact of sex effects on cancer-nervous system interactions.

To investigate the sex-specific effects of these interaction DMPs, we directly compared the DNAm changes of these loci in a sex-stratified manner. We found that the effect sizes of these interaction DMPs were significantly sex-biased in six out of nine cancers (Fig. 4d and Supplementary Fig. 2f). Specifically, THCA (Slope, *S* = 3.1, FDR < 0.001), COAD (*S* = 2.8, FDR < 0.001), HNSC (*S* = 2.3, FDR < 0.001), BLCA (*S* = 1.9, FDR = 0.039), KIRC (*S* = 1.8, FDR = 0.032), and LUAD (*S* = 1.5, FDR = 0.002) showed larger magnitude of changes in males than in females. These observations are consistent with the sex-stratified results, highlighting that sex effects on cancers may largely manifest through differences in the magnitude of multiple DNAm effects.

### Amplification effects of DNAm account for sex differences in cancers

Our observations highlight a pervasive sex difference in the magnitude of DNAm effects on cancers. However, involving dysregulation of multiple genes during cancer progression, how to quantify the extent of sex-dependent effects in cancers presents a challenge. Inspired by a recent study [35] that proposed sex differences in the magnitude of many genetic effects (‘‘amplification’’) as the primary mode of gene-by-sex interaction in complex human traits, we inferred that such a theory could be extended to DNAm levels in cancers. To evaluate the sex-dependent amplification effects in cancers, we examined the covariance relationships of DNAm effects between females and males using the multivariate adaptive shrinkage (mash) approach (Methods).

**Fig. 5:**
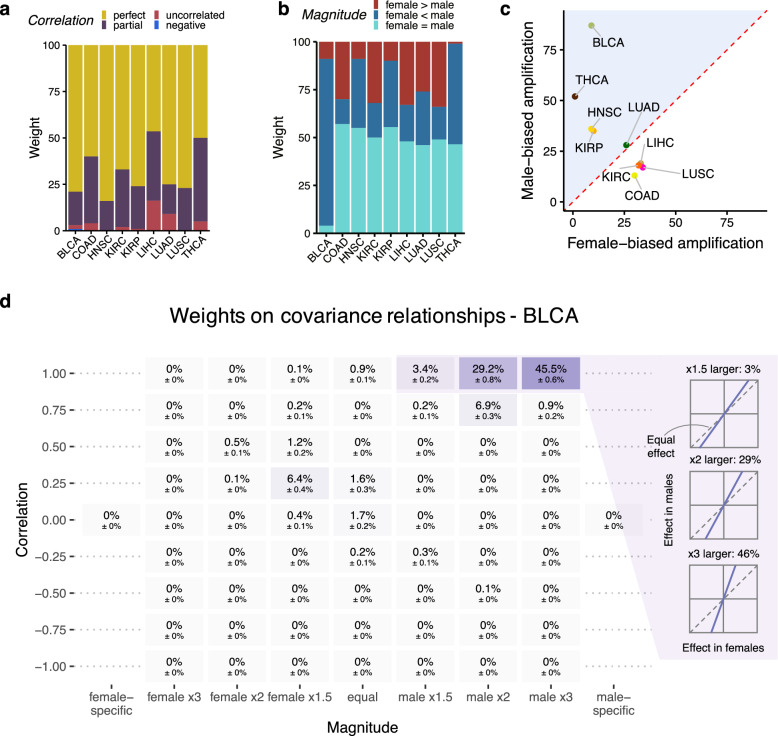
Amplification effects on DNAm in cancers. **a** Proportions of weights on different types of correlation relationships between female and males for each cancer. Perfect correlation is indicated when corr = 1. Partial correlation is represented when 0 < corr < 1. Negative correlation is denoted when corr < 0. Uncorrelated condition is described when corr = 0.** b** Proportions of weights on different types of magnitude relationships between sexes for nine cancers. "female > male" indicates larger effect sizes in females than in males. "female < male" indicates larger effect sizes in males than in females. "female = male" represents equal effect sizes between sexes. **c** The difference between the fraction of male-larger effects and the fraction of female-larger effects classified these nine cancer types into female- and male-biased group. The male-biased group is located above the diagonal, while female-biased group is located below the diagonal. **d** Example of weights on covariance matrices for BLCA, which exhibited the greatest magnitude of DNAm changes in males

For these nine cancer types, we noticed that the DNAm effects exhibiting negative correlation (corr < 0) were more likely to be marginal effects, unable to survive the multiple testing correction penalty (Supplementary Fig. 3b). On average, 68% of DNAm effects showed perfect correlation (corr = 1) between sexes in cancers, with an additional 27% exhibiting partial correlation (0 < corr < 1) (Fig. 5a, Supplementary Fig. 3d and Supplementary Table 9). These suggest that most DNAm effects were perfectly or partially correlated. While negative correlation, representing opposite regulation of DNAm between sexes, may not be the primary driver of sex differences in cancers.

In terms of magnitude relationships, we found widespread sex-biased effect sizes across cancers. Specifically, on average, 34% of effects exhibited male-biased larger DNAm changes across the nine cancers, while 20% exhibited female-biased larger changes (Fig. 5b, Supplementary Fig. 3d and Supplementary Table 9). These findings align with our sex-stratified results, indicating that the cancer-related DNAm effects between sexes were largely correlated but with varying magnitudes.

We next classified these nine cancer types based on the difference between the fraction of male- and female-larger effects. The female-biased group included LUSC, LIHC, COAD, and KIRC, each exhibiting a relatively greater percentage of female-larger effects (Fig. 5c, Supplementary Fig. 3c and Supplementary Table 9). The male-biased group included BLCA, THCA, KIRP, LUAD, and HNSC, each showing a relatively higher percentage of male-larger effects (Fig. 5c, Supplementary Fig. 3c and Supplementary Table 9). Remarkably, BLCA displayed the greatest magnitude of DNAm changes in males. Of the 79% weights on matrices showing perfect correlation between sexes, 78% of the weights represented larger DNAm changes in males, with 46% exhibiting three times male-larger effects (Fig. 5d, Supplementary Fig. 3d and Supplementary Table 9). Together, these observations point to amplification effects of DNAm as the primary mode for sex differences in cancers.

### DNAm signals exhibiting cancer-related sex amplification effects

**Fig. 6:**
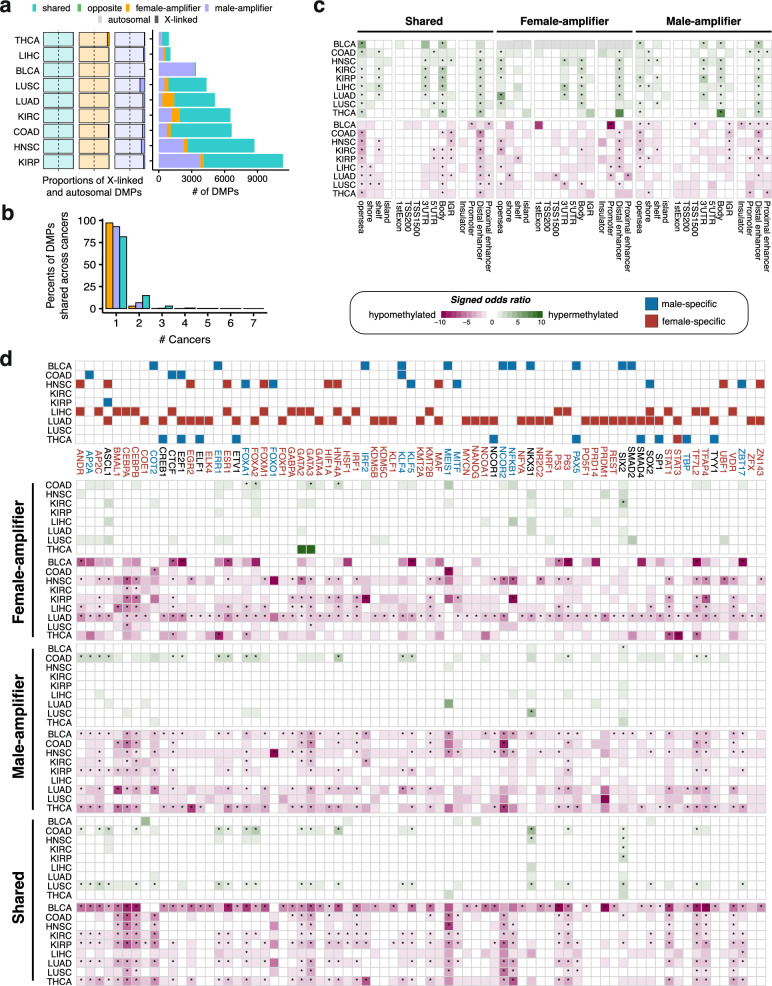
Prioritize DNAm signals exhibiting cancer-related sex amplification effects. **a** The number of cancer-related DMPs exhibiting the sex amplification effects (right, histogram). No significant opposite effects between sexes were identified. Proportions of X-linked and autosomal DMPs for sex-shared effects, female-amplifiers, and male-amplifiers (left). **b** Cancer sharing profile of sex-shared effects, female-amplifiers, and male-amplifiers. **c** Enrichment of genomic functional elements (CGI regions, genomic features, and cCRE regions) for sex-shared effects and sex-amplifiers. The signed odds ratios are labeled in colors (two-sided: hypermethylated and hypomethylated). Significant FDR-corrected *P*-values are indicated (*FDR < 0.05). **d** Sex-specific TFBS enrichment for sex-shared DMPs and sex-amplifiers. The TFs that were sex-specifically enriched in cancers are depicted (top). The TFBS enrichment profiles for each cancer are shown (bottom). The red words represent TFs with female-specific TFBSs enrichment, blue words represent TFs with male-specific TFBSs enrichment, and black words represent TFs with sex-biased TFBSs enrichment. The signed odds ratios are labeled in colors (two-sided: hypermethylated and hypomethylated). Significant FDR-corrected *P*-values are indicated (*FDR < 0.05). **e** The heatmaps illustrate the top three significantly estimated hazard ratios (HRs) for each cancer, which are associated with overall survival. *P < 0.05; **P < 0.01; ***P < 0.001. *CGI* CpG island, *cCREs* candidate cis-regulatory elements, *TFBSs* transcription factor binding sites

We then prioritized the hub DNAm signals representing the observed magnitude differences in cancer-related DNAm changes across sexes. We assessed the heterogeneity in effect sizes between sexes for each cancer and integrated these results with sex-stratified findings and posterior estimates assigned by the mash model (Supplementary Fig. 4; Methods). These analyses yielded 3,361 female-amplifiers, 11,837 male-amplifiers, and 25,911 sex-shared DNAm effects across nine types of cancers (Fig. 6a and Supplementary Table 10). No significant opposing effects between sexes were identified (Fig. 6a). These DNAm effects were largely (> 81%) cancer-specific (Fig. 6b).

To determine the functional regulatory role of these sex-shared and sex-amplified effects in cancers, we performed enrichment analysis of genomic functional elements for these effects. We found that these effects were both significantly enriched in gene regulatory regions, particularly in gene body regions, untranslated exon regions (UTRs), and distal enhancer regions (Fig. 6c and Supplementary Table 11).

DNAm in enhancer regions is closely linked to regulating gene expression. We inferred that could sex-specifically affect the occupancy of transcription factors (TFs) at their binding sites (TFBSs). To test this, we examined the enrichment of 137 TFs for their binding sites in sex-shared and sex-amplified effects. We found that the enrichment for TFBSs were largely driven by hypomethylated DMPs (Fig. 6d and Supplementary Table 12). Among the tested TFs, 54.74% (75/137) were sex-specifically enriched in at least one cancer type, with TFBSs for 24 TFs implicated in two types (Fig. 6d). Such is the case of *EGR2,* which was specifically enriched in female-amplifiers of both LUAD and HNSC (Fig. 6d). A recent study highlighted the role of *EGR2* in coordinating the alveolar macrophage functional program [56]. Our findings imply a female-specific role of *EGR2* in LUAD under the influence of female-amplified aberrant DNAm. Moreover, 14 TFs displayed sex-biased roles in different cancers according to the enrichment profiles (Fig. 6d and Supplementary Table 12). For instance, *CTCF* was significantly enriched in male-amplifiers in COAD, whereas in female-amplifiers in LIHC. Additionally, to identify enriched conserved motifs within the X-linked sex-amplifiers in cancers, we used HOMER [48]. Our analysis revealed enrichment for three conserved TF motifs among the X-linked female-amplifiers and 66 motifs for the male-amplifiers (Supplementary Table 12). Notably, 17 TFs among the X-linked male-amplifiers exhibited motif enrichment across more than two cancer types, including AP-1, E2F1, and Sox7. Importantly, the enriched motifs for female- and male-amplifiers were distinct.

To understand the biological roles of sex-amplified DMPs in cancers, we performed functional enrichment analyses. For female-amplifiers, we observed dysregulation of immune-related pathways, including interleukin-18-mediated signaling in LIHC, type I interferon signaling in HNSC, and antigen presentation via MHC class II in BLCA (Supplementary Fig. 5a and Supplementary Table 13). In male-amplifiers, pathways related to cell proliferation, migration, and TGF-β signaling were affected, such as negative regulation of endothelial cell proliferation in LUSC, regulation of cell migration in KIRC and THCA, and response to TGF-β in BLCA (Supplementary Fig. 5b and Supplementary Table 14).

Given the pervasive enrichment of synapse-related pathways among AMPs, we examined sex-amplified genes within synaptic biological functions using the SynGO database [57]. We found that 244 female-amplified genes and 540 male-amplified genes were annotated with synaptic functions. Although synaptic process enrichment is broadly consistent between sexes, differences exist in the specific biological terms and genes mapped within the SynGO hierarchy (Supplementary Fig. 5c and Supplementary Table 15). For example, male-amplified genes such as *DLG3*, *PORCN*, and *PPFIA1* are involved in regulating postsynaptic membrane neurotransmitter receptor levels across multiple cancer types. In contrast, a distinct set of female-amplified genes, such as *NRXN3* and *EFNB2*, are linked to the same pathway in comparable cancer types. Additionally, male-amplified genes *EXOC2*, *ATG16L1*, and *NETO1* are associated with axonal transport in BLCA, HNSC, COAD, and KIRP, indicating potential male-specific pathological mechanisms. The diversity of synaptic functions among sex-amplified genes implies that various functional interactions between sex and cancer risk converge at the synapse.

To demonstrate the functional and clinical relevance of these DNAm divergences, we assessed the association of sex-amplifiers with overall patient survival and recurrence following initial treatment. Using Lasso regression for variable selection followed by multivariate Cox analysis, we identified 24 female-amplifiers and 29 male-amplifiers that were sex-specifically associated with overall survival (Fig. 6e and Supplementary Table 15). For example, the female amplifier for BLCA, cg19395441, was significantly associated with overall survival in females (HR = 1.48, 95% CI 1.21–1.82, *P* < 0.001) but not in males (HR = 0.96, 95% CI 0.86–1.07, *P* = 0.48). Additionally, we identified 15 female-amplifiers and 13 male-amplifiers associated with recurrence (Supplementary Table 15). These findings highlight the potential of identified sex-amplifiers as the sex-specific prognostic biomarkers.

### Integration of DNA methylation and gene expression highlights sex-dependent regulation and biological processes

**Fig. 7:**
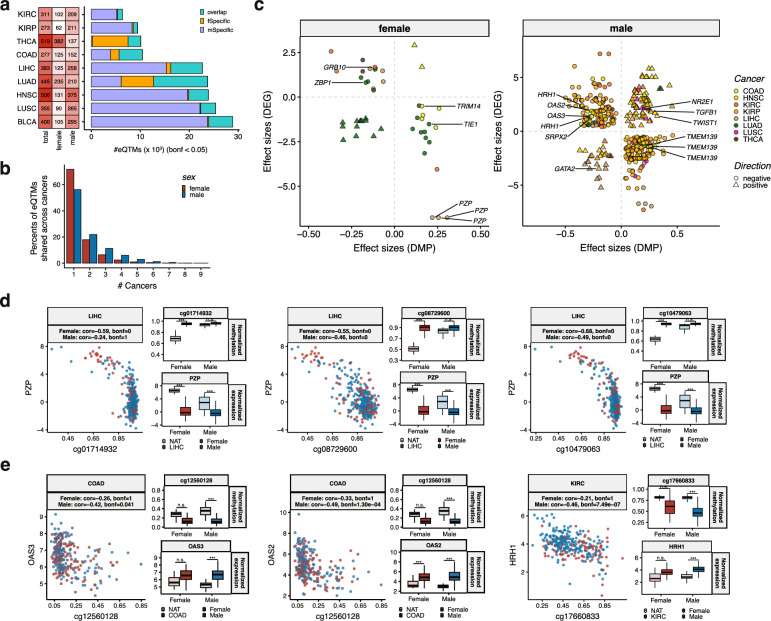
Characterizing the correlations between DNAm and gene expression. **a** Discovery of eQTMs pairs per sex and per cancer. Left, the number of sample sizes per sex and per cancer (heatmap). Right, the number of eQTMs (Bonferroni-adjust *P* < 0.05, histogram). The sex-overlapped eQTMs, female-specific eQTMs, and male-specific eQTMs are indicated. **b** Cancer sharing profile of eQTMs per sex. **c** Discovery of the detected sex-amplifiers with significantly sex-stratified differential gene expression using eQTM pairs. The directions of correlation are depicted. **d** Examples of female-amplifiers that were significantly correlated with gene expression of target genes, exhibiting a consistently greater magnitude of gene expression changes in females compared to males. **e** Examples of male-amplifiers that were significantly correlated with gene expression of target genes, exhibiting a consistently greater magnitude of gene expression changes in males compared to females. *P_*bonf*_ < 0.05; **P_*bonf*_ < 0.01; ***P_*bonf*_ < 0.001. *eQTMs* methylome and transcriptome correlations

The primary function of DNAm is to regulate gene expression. To gain insight into its potential regulatory role, we characterized the correlations between methylome and transcriptome (eQTMs) in each sex per cancer, using matched samples. A total of 38,969 eQTMs were identified in females, ranging from 1,135 in KIRP to 17,685 in LUAD (Spearman’s *P*_*bonf*_ < 0.05, Fig. 7a and Supplementary Table 16). Meanwhile, 77,930 eQTMs were observed in males, ranging from 2,770 in THCA to 28,605 in BLCA (Spearman’s *P*_*bonf*_ < 0.05, Fig. 7a and Supplementary Table 16). On average, 24.17% of eQTMs were shared between sexes with consistent directions of correlation (Fig. 7a), and none of the eQTMs exhibited opposite correlations between sexes. Among these eQTMs, 71.74% (27,958/38,969) of female-related eQTMs and 56.19% (43,788/77,930) of male-related eQTMs were significantly correlated in only one type of cancer, indicating cancer-dependent regulation (Fig. 7b). The prevalence of sex-specific eQTMs across cancers highlights extensive sex-dependent regulatory mechanisms.

To investigate the functional mechanisms underlying these eQTMs, we performed enrichment analysis of candidate cis-regulatory elements (cCREs) for eCpGs in negatively and positively correlated eQTMs per sex, respectively. A similar enrichment pattern was observed in female- and male-related eQTMs. In both sexes, positively correlated eQTMs were more frequently enriched in distal enhancer regions and insulators, whereas negatively correlated eQTMs were predominantly enriched in promoters and proximal enhancer regions (Supplementary Fig. 6a and Supplementary Table 17).

We next prioritized eQTM pairs associated with sex-amplified DMPs. For female-amplifiers, 48 eQTMs were extracted across five types of cancers (number of eQTMs in descending order; LUAD: 27, LIHC: 8, COAD: 7, KIRC: 4, and THCA: 2, Fig. 7c and Supplementary Table 18). These eQTMs comprised 44 eCpGs and 42 eGenes, with a majority (68%, 33/48) exhibiting negative correlations (Fig. 7c). Consistent with DNAm patterns, 32 eGenes demonstrated greater magnitudes of expression changes in females compared to males (Supplementary Table 18). Notably, in LIHC, three female-amplifier CpGs (cg01714932, cg08729600, and cg10479063) were significantly negatively correlated with *PZP* gene expression (Spearman’s *P*_*bonf*_ < 0.05), with larger expression changes in females (Fig. 7d and Supplementary Table 18). Additional notable eQTM pairs for female-amplifiers included cg17306740-*ZBP1* in LUAD, cg01765174-*TRIM14* in COAD, and cg25748357-*GRB10* in KIRC (Supplementary Fig. 6b-d and Supplementary Table 18).

Regarding the male-amplifiers, 380 eQTMs were extracted for eight cancer types linked to 356 eCpGs and 272 eGenes (number of eQTMs in descending order; KIRP: 183, HNSC: 68, KIRC: 40, LUSC: 34, LIHC: 27; COAD: 15, LUAD: 12, THCA: 4, Fig. 7c and Supplementary Table 18). Of these, 277 eQTMs exhibited negative correlations (Fig. 7c), with 285 showing consistently greater magnitudes of DNAm and gene expression changes in males. For instance, the male-amplifier cg12560128 was negatively correlated with *OAS2* and *OAS3* in COAD (Fig. 7e and Supplementary Table 18), both known immune biomarkers linked to the tumor microenvironment [58, 59]. Additionally, *HRH1*, which correlated negatively with cg17660833 in KIRC, has been implicated in T cell dysfunction and is frequently upregulated in the tumor microenvironment [60]. (Fig. 7e and Supplementary Table 18). Additional examples include cg06490988-*GATA2* in KIRP, cg19193956-*TWIST1* in HNSC, cg13784235-*NR2E1* in LUSC and cg15652212/cg22531018/cg21852589-*TMEM139* in LIHC (Supplementary Fig. 6e-j and Supplementary Table 18). These results suggest that sex-amplified DNAm changes can influence downstream gene expression, contributing to consistent sex-biased differential expression in cancers.

To elucidate the biological roles of genes regulated by sex-amplifiers through eQTMs, we performed functional enrichment analysis. Our findings indicate that genes regulated by male-amplifiers are associated with cancer development-related pathways, including vasculature development, epithelial cell differentiation, and myeloid cell differentiation (Supplementary Fig. 7 and Supplementary Table 19). In contrast, genes regulated by female-amplifiers are linked to specific pathways, such as sprouting angiogenesis in COAD and cell communication in THCA (Supplementary Fig. 7 and Supplementary Table 19).

We further evaluated the therapeutic potential of genes regulated by sex-amplifiers through eQTMs. Using druggable targets annotated in the CIViC database [53], we found that several genes were previous identified as drug targets (Supplementary Table 18). For instance, *ECSCR* in LUAD and *PTK2B* (also known as *PYK2*) in COAD were regulated by female-amplifiers, while *GATA2* in KIRP, *ERBB3* in HNSC, and *POU5F1* in KIRC were regulated by male-amplifiers. These observations indicate that these previously identified druggable targets may have a sex-specific role in cancers.

## Discussion

Based on these DNAm-focused integrative analyses, the findings presented here substantially refine our understanding of sex differences in cancers. Our analyses revealed a notable attenuation of sex differences in DNAm within cancer tissues compared to paired NATs. Using a sex-stratified case–control approach, we uncovered widespread and largely cancer-specific sex-dependent dysregulation of DNAm across various cancers. Furthermore, comparing the covariance relationships of cancer-related DNAm changes between sexes revealed that the amplification effect of DNAm contributes to the observed sex differences. Characterization of the gene expression regulation of DNAm through eQTMs showed that sex-amplified DNAm variations in cancer could influence the expression of target genes, leading to consistent sex-biased differential expression. Collectively, these results provide critical insights into the mechanisms driving sex differences in DNAm within cancers.

Sex differences in the genomics of healthy individuals and cancers have been previously revealed [9–12]. However, the lack of studies leveraging the sex differences between these two conditions has left it unclear whether such disparities are reorganized during cancer progression. By comparing with paired NATs, we observed a striking reduction of sex differences in DNAm across cancers, resulting in a more epigenetically homogeneous pattern between sexes within cancers. These findings underscore the importance of including matched NAT samples when examining sex effects in cancers. As expected, the AMPs, which exert their influence through the attenuating role of sex-related differential DNAm, were primarily X-linked. Remarkably, we found that these X-linked AMPs, with larger methylation levels in females, were mostly located in the conserved regions of X chromosomes, where genes typically get inactive. This suggests a potential mechanism for the reorganization of DNAm patterns within X chromosome during cancer progression, likely driven by escape from XCI. Surprisingly, a significant enrichment of synapse-related pathways in AMPs was identified in a wide range of cancers, including LUAD, LUSC, BLCA, COAD, and KIRP. Additionally, we observed a diverse range of synaptic functions among interaction DMPs and sex-amplified genes across cancers. Recent studies have highlighted the critical role of both the peripheral and central nervous systems in regulating tumorigenesis and metastasis [61, 62]. Our findings extend the burgeoning field of cancer neuroscience into a sex-dependent context, suggesting that various functional interactions between sex and cancer risk converge at the synapse.

The imbalance of sex differences in DNAm between cancer and NATs raises the question of whether the extent of DNAm alteration in cancers differed by sex. By applying a sex-stratified case–control strategy and investigating the covariance relationships between sexes, we found that the identity and direction of differential methylation were largely shared between sexes, resulting in highly correlated effect sizes. However, the magnitude of DNAm changes differed. These observations align with a recent study that proposed amplification as the primary mode of gene-by-sex interaction in human complex traits [35]. Our work extends this theory to the disease level of cancer. Despite mixed covariance relationships of DNAm effects across sexes within each cancer types, we noticed that these mixture weights could be concentrated on one sex. By analyzing the ratios of male-to-female weights, we categorized these nine cancer types into male-biased (BLCA, THCA, KIRP, LUAD, and HNSC) and female-biased (LUSC, LIHC, COAD, and KIRC) groups. Particularly notable was the striking concentration of mixture weights for BLCA on the male-biased magnitudes. Previous research defined weak and strong sex-effect groups based on sex-biased molecular signatures within cancer tissues [9]. We observed that eight of the cancers in our analysis (BLCA, THCA, KIRP, LUAD, HNSC, LUSC, LIHC, and KIRC) align with the previous defined strong sex-effect groups [9]. It indicating that our approach to studying sex effects on DNAm in cancers advances previous classifications into a sex-specific scenario, which could facilitate the understanding of how sex effects contribute to different cancer types.

Our study reported a series of DMPs exhibiting sex-amplified roles in different cancers and noted cancer specificity in the enrichment of TFBSs for these sex-amplifiers. This enrichment was largely driven by hypomethylated DMPs. Loss of DNAm at TFBSs can active specific TF networks during reprogramming and cancer progression [63, 64], indicating sex-dependent regulation involved. Notably, among the 14 TFs whose binding sites were sex-dependently enriched across different cancers, eight were previously found to be enriched among sex-biased expressed genes, including *SP1*, *ETV1*, *ELF1*, *E2F1*, *CREB1*, *CTCF*, *SIX2*, and *SOX2* [11]. Moreover, several TFs play known roles in cancers, such as *ASCL1* [65, 66], *NCOR1* [67], *SMAD2* [68], and *SMAD4* [68]. However, the interaction between sex and cancer on most TFs remains uncharacterized. Importantly, TFBSs enrichment in sex-amplifiers is not driven by sex-biased methylation of the TFs themselves. Additionally, genes related to sex amplifier-related eQTMs were not those TFs whose binding sites were enriched in sex-amplifiers. Our findings align with the observation that sex-biased TF targeting of genes is independent of sex-biased gene expression [11, 12]. These findings indicate that the interplay between sex and cancer may act through influence on the binding sites of TFs by sex-amplified DNAm dysregulation. DNAm regulation in cancers is sex-biased, and further investigation is warranted to verify these findings, as observed patterns may originate from a variety of mechanisms.

Notably, our analysis revealed that sex-amplified DMPs exhibit higher cancer specificity compared to sex-stratified DMPs. Specifically, 93% of male-amplified DMPs and 97% of female-amplified DMPs were exclusive to one single cancer type, significantly exceeding the cancer specificity observed for sex-stratified DMPs, which were 77.41% for females and 67.07% for males. One plausible explanation for this observation is that sex-amplified DMPs represent DNA methylation divergences characterized by substantial differences in effect size between sexes, which are accentuated in specific cancer contexts. This reflects the unique biological mechanisms underlying sex-biased DNA methylation changes that amplify in the context of particular cancers, driven by both sex- and cancer-specific factors. Consequently, the pronounced cancer specificity of sex-amplified DMPs underscores their potential as highly precise biomarkers for cancer diagnosis and prognosis, emphasizing their clinical relevance.

Several limitations should be noted. The datasets obtained from TCGA were not initially designed to investigate sex effects in cancers, resulting in an imbalance of samples sizes between females and males. In this study, rigorous methodology was used to account for biological and technical variability, ensuring that the results reported here are conservative and generalizable. Further studies are still needed to increase the sample size and validate the key findings. Additionally, we acknowledge the limited statistical power for survival analysis for recurrence in KIRC, which is due to the availability of data for only two patients in TCGA. This limitation may be attributed to the relatively favorable prognosis of KIRC or potential information loss during follow-up. Moreover, to better decipher the crosstalk between sex effects and cancer progression, employing analyses of individual changes over time will be necessary for further refinement of the results presented here.

## Conclusion

By systematically comparing the sex differences between cancers and NATs, our research provides a comprehensive overview of the DNAm landscape with respect to sex differences in cancers. Our study revealed a significant attenuation of sex differences in cancers and highlighted that amplification effects of DNAm predominantly drive the observed sex differences during cancer progression. These findings substantially advance our understanding of the interplay between DNAm and sex in cancer etiology. Additionally, our study identifies promising candidate CpGs and genes for further sex-specific functional characterization and drug development.

## Supplementary Information

Supplementary material 1.

Supplementary material 2.

Supplementary material 3.

Supplementary material 4.

Supplementary material 5.

Supplementary material 6.

Supplementary material 7.

Supplementary material 8.

Supplementary material 9.

Supplementary material 10.

Supplementary material 11.

Supplementary material 12.

Supplementary material 13.

Supplementary material 14.

Supplementary material 15.

Supplementary material 16.

Supplementary material 17.

Supplementary material 18.

Supplementary material 19.

Supplementary material 20.

## Data Availability

The DNAm and RNA-seq data analyzed here are available through the TCGA Research Network (https://tcga-data.nci.nih.gov/tcga/) and through GEO under accession codes GSE54503, GSE66836, GSE199057, GSE61441, and GSE97466. All data are available in the main text or the supplementary materials. The code of this work can be found at https://github.com/zhoujiaqi704/sex_differences_cancers.git.
