# Supplementary material for: Attenuated sex-related DNA methylation differences in cancer highlight the magnitude bias mediating existing disparities
Source: Biol Sex Differ. 2024 Dec 23;15:106. doi: 10.1186/s13293-024-00682-4 (PMC11664931; doi:10.1186/s13293-024-00682-4)
Supplement: Supplementary file 1 — Supplementary material 1. [file 13293_2024_682_MOESM1_ESM.pdf]

Supplementary Figures

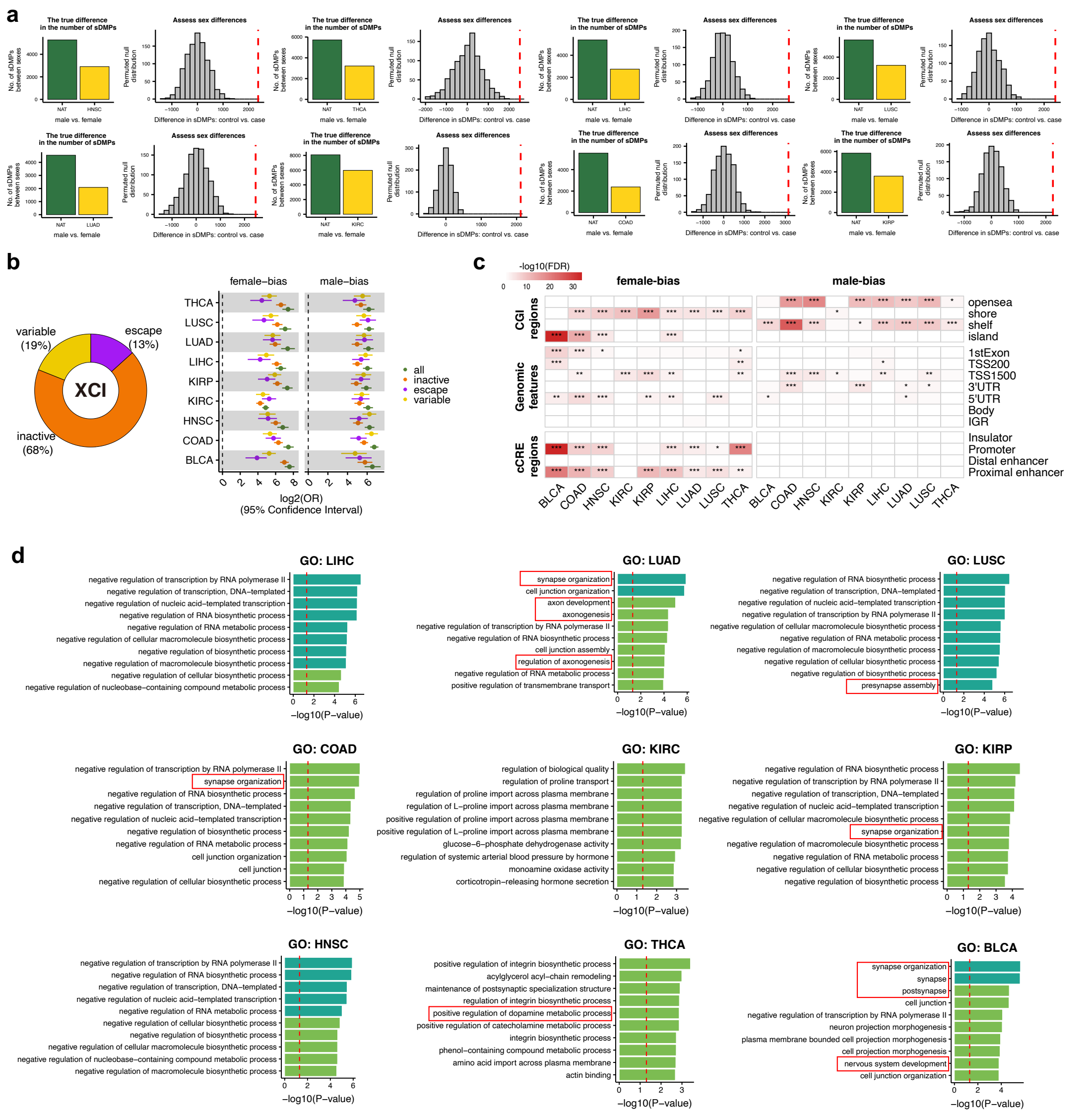

Supplementary Fig. 1 Attenuation of sex differences in DNAm levels cross cancers. a, identifying statistically significant differences in the number of sDMPs across eight cancers, except BLCA. The histogram depicts the true difference in the number of sDMPs between BLCA and NAT samples. The density plot depicts the permuted null distribution (1000 times), which is then used to determine the significance of the difference in sDMPs between BLCA and NAT samples. **b**, On the left, the donut plot depicts the percentage of AMP genes that were previously defined XCI genes. On the right, it represents the enrichment of female- and male-biased AMP genes in XCI genes. **c**, Enrichment of genomic functional elements (CGI regions, genomic features, and cCRE regions) for female- and male-biased AMPs. *FDR < 0.05, **FDR < 0.01, ***FDR < 0.001. **d**, Top ten GO annotations for AMPs per cancer. Dark green represents the FDR < 0.05, while light green indicates nominal $P < 0.05$. DNAm, DNA methylation; sDMPs, sex-related differentially methylated positions; BLCA, bladder urothelial carcinoma; NAT, normal adjacent tissues; AMPs, attenuated sDMPs; XCI, X chromosome inactivation; CGI, CpG island; cCRE, candidate cis-regulatory elements; GO, Gene Ontology.

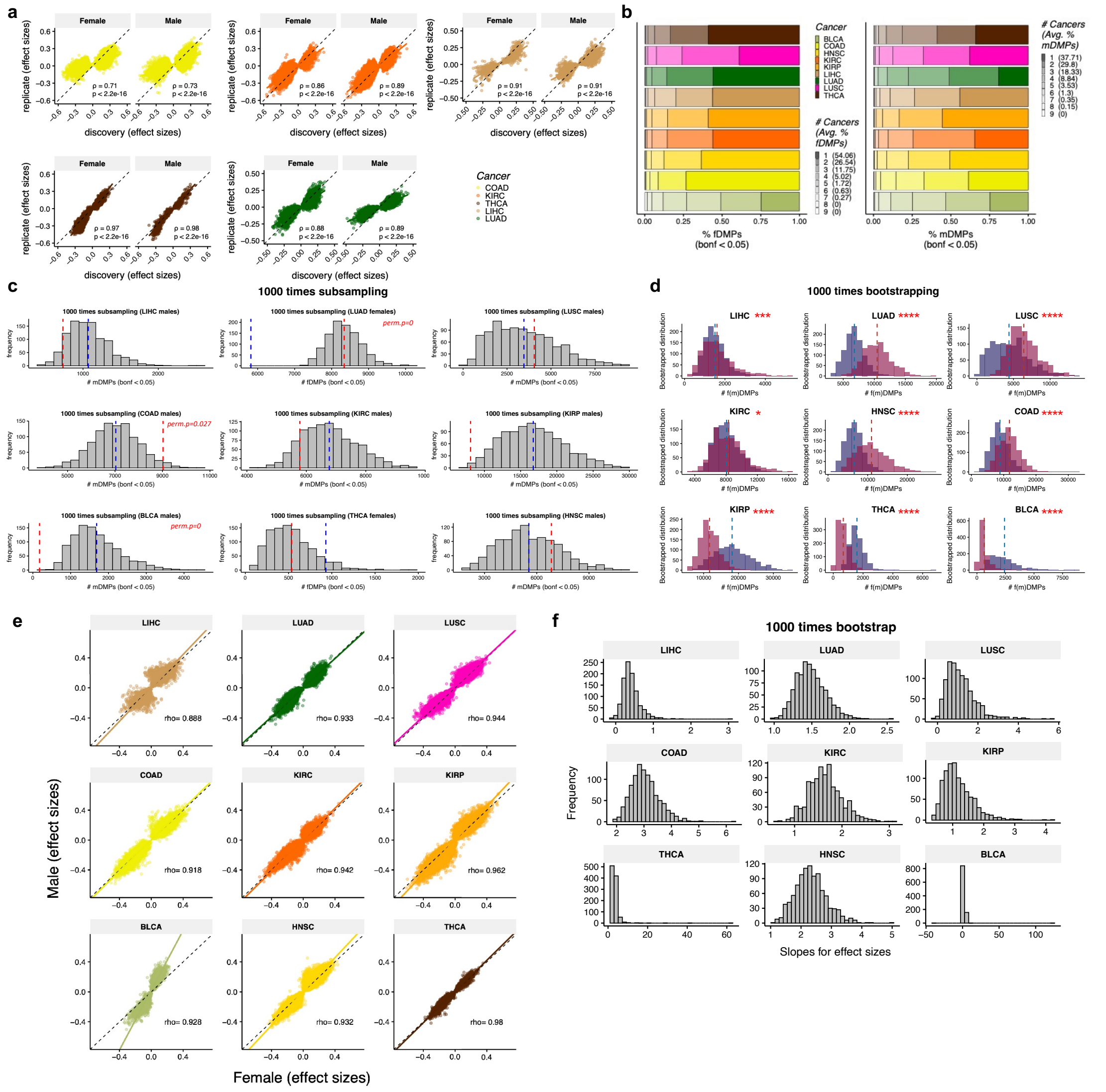

Supplementary Fig. 2 Sex effect on cancer-related differential DNAm. a, Correlation of effect sizes between discovery data and replicate data for the fDMPs and mDMPs identified in discovery datasets. **b**, Cancer-sharing profiles of fDMPs and mDMPs for each cancer types. **c**, Down-sampling analyses to account for differences in sample size across sexes, using permutation p-value to represent the significance of differences in DMP numbers between sexes when the sample size are matched. For males with larger sample sizes (LIHC, LUSC, COAD, KIRC, KIRP, HNSC, and BLCA), the red dashed line represents the true number of fDMPs, while the blue dashed line represents the mean number of mDMPs. For females with larger sample sizes (LUAD and THCA), the red dashed line represents the mean number of fDMPs, while the blue dashed line represents the true number of mDMPs. **d**, Bootstrapping approach through sampling subjects with replacement was used to compare the distribution of the number of fDMPs and mDMPs. The red dashed line represents the mean number of fDMPs, while the blue dashed line represents the mean number of mDMPs. The significance of differences in fDMPs and mDMPs was calculated using Wilcoxon rank sum tests ($P < 0.05$ as the significant threshold). **e**, Effect sizes correlation of sex-stratified DMPs between female and male subgroups. **f**, Bootstrapping distribution of slopes of effect sizes of these interaction DMPs between sexes. fDMPs, female-related DMPs; mDMPs, male-related DMPs.

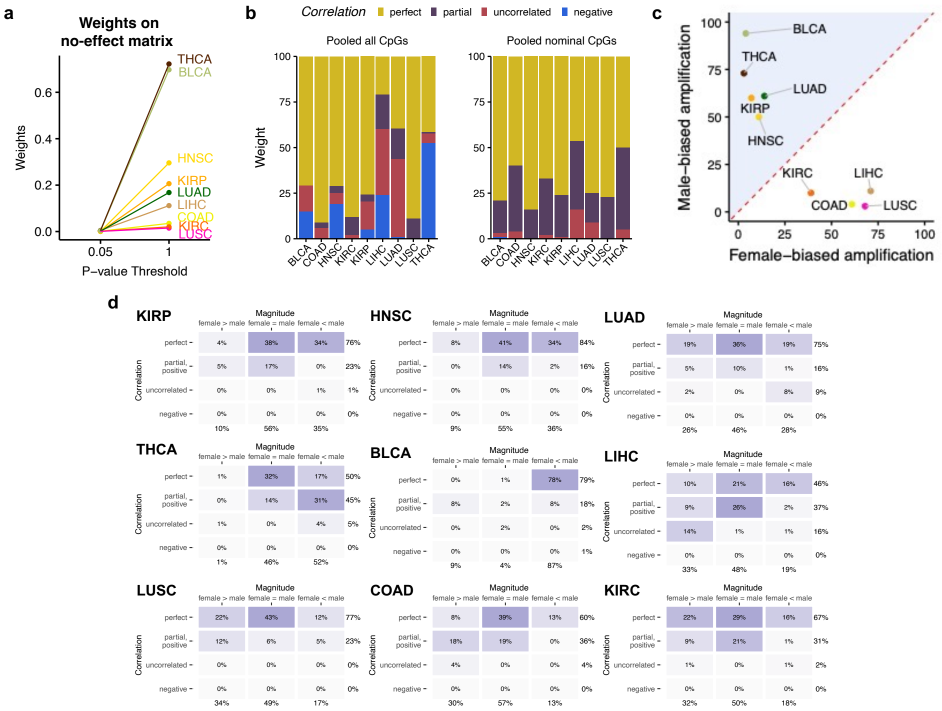

Supplementary Fig. 3 Amplification of DNA methylation effects contribute to sex differences in cancers. a, The weights on the no-effect matrix at two different *p*-value thresholds (all tested CpGs with a *p*-value ≤ 1; nominal CpGs with a *p*-value < 0.05). **b**, Correlation relationships of effect sizes between females and males. Perfect correlation is indicated when corr = 1. Partial correlation is represented when 0 < corr <1. Negative correlation is denoted when corr < 0. Uncorrelated condition is described when corr = 0. **c**, The difference between the fraction of male-larger effects and the fraction of female-larger effects based on all CpGs, classifying these nine cancer types into female- and male-biased group. The male-biased group is located above the diagonal, while female-biased group is located below the diagonal. **d**, The weights on covariance matrices for all nine cancers.

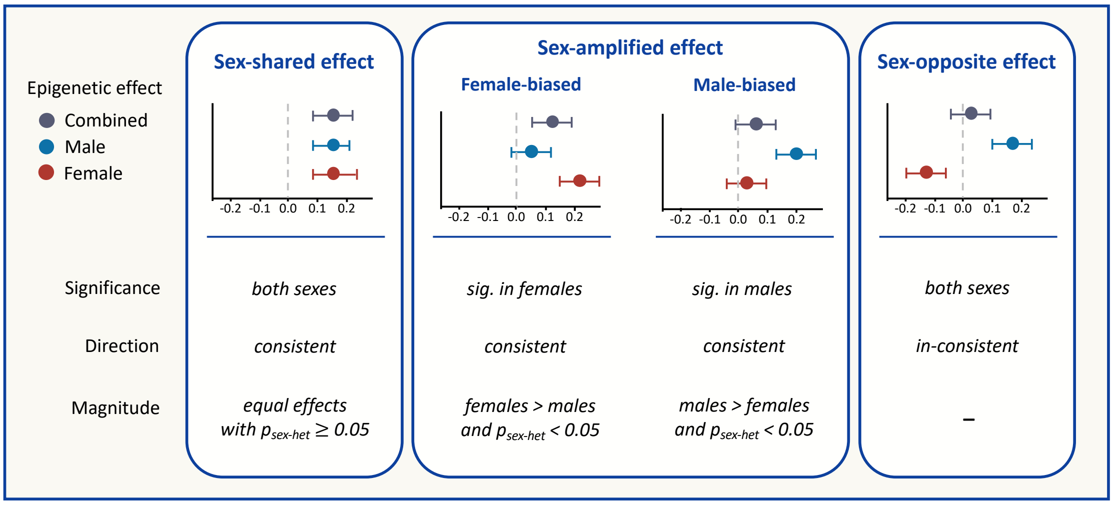

Supplementary Fig. 4 The criteria for sex-effect DMPs categorization. The sex-stratified sDMPs were classified into four main types, including sex-shared effects, female-amplifiers, male-amplifiers and sex-opposite effects.

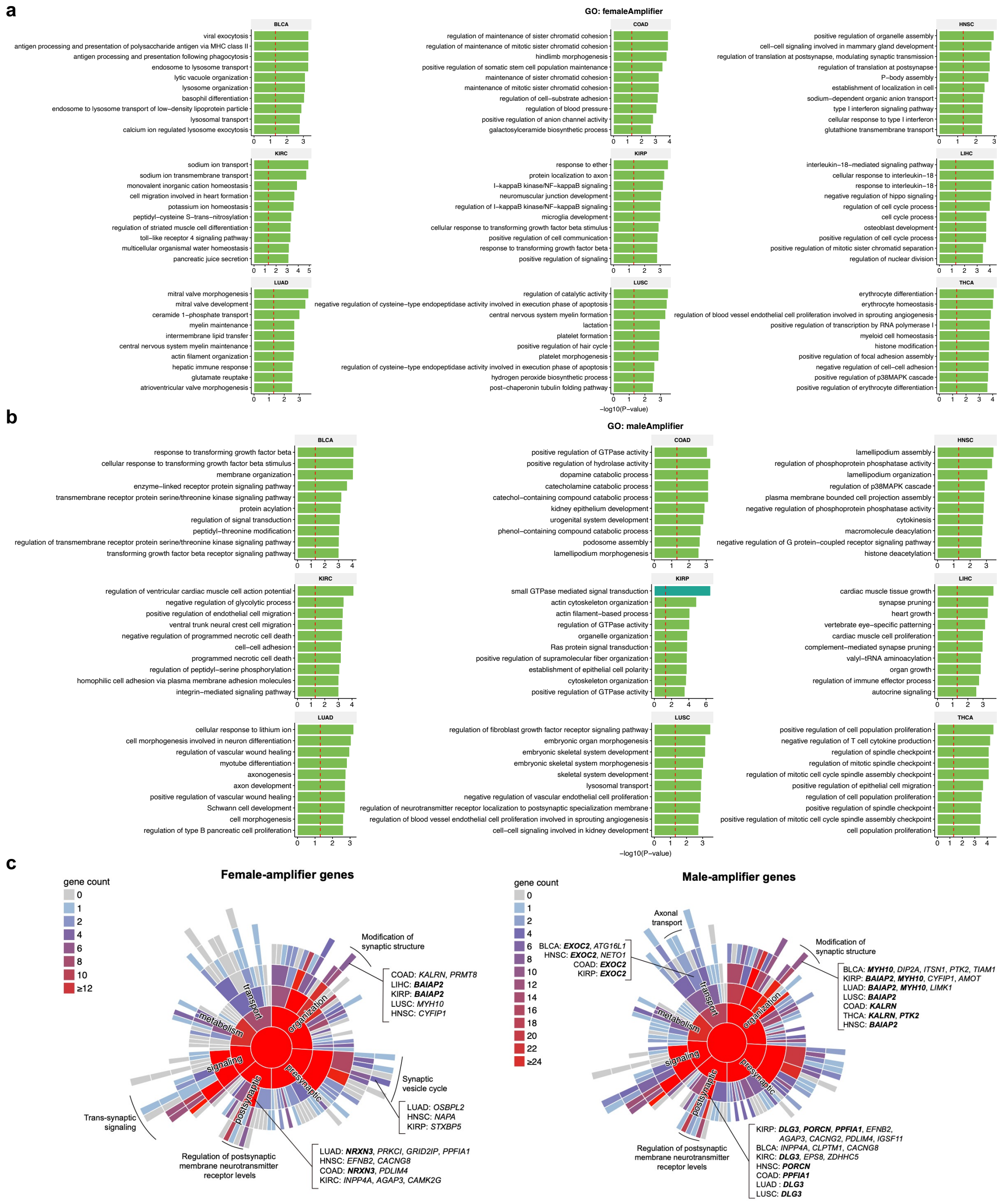

Supplementary Fig. 5 DNA methylation signatures underlying cancer-related sex amplification effects. a and b, Top ten GO annotations (focusing on BP terms) for (**a**) female-amplifiers and (**b**) male-amplifiers per cancer. Dark green represents FDR < 0.05, while light green indicates nominal *P* < 0.05. **c**, Sunburst plots illustrate synapse-related biological processes, beginning with the synapse at the center. The first ring represents categories such as synaptic signaling, metabolism, transport, organization, and processes in the pre- and postsynaptic regions, followed by child terms (subsets of the adjacent inner ring) in the outer rings. The number of genes associated with each term is indicated using the color scheme shown in the legend. (Left) Female-amplified genes: 244 are annotated in SynGO, with 182 mapped to biological processes. (Right) Male-amplified genes: 540 are annotated in SynGO, with 395 mapped to biological processes. Genes that are shared across more than two cancer types are shown in bold.

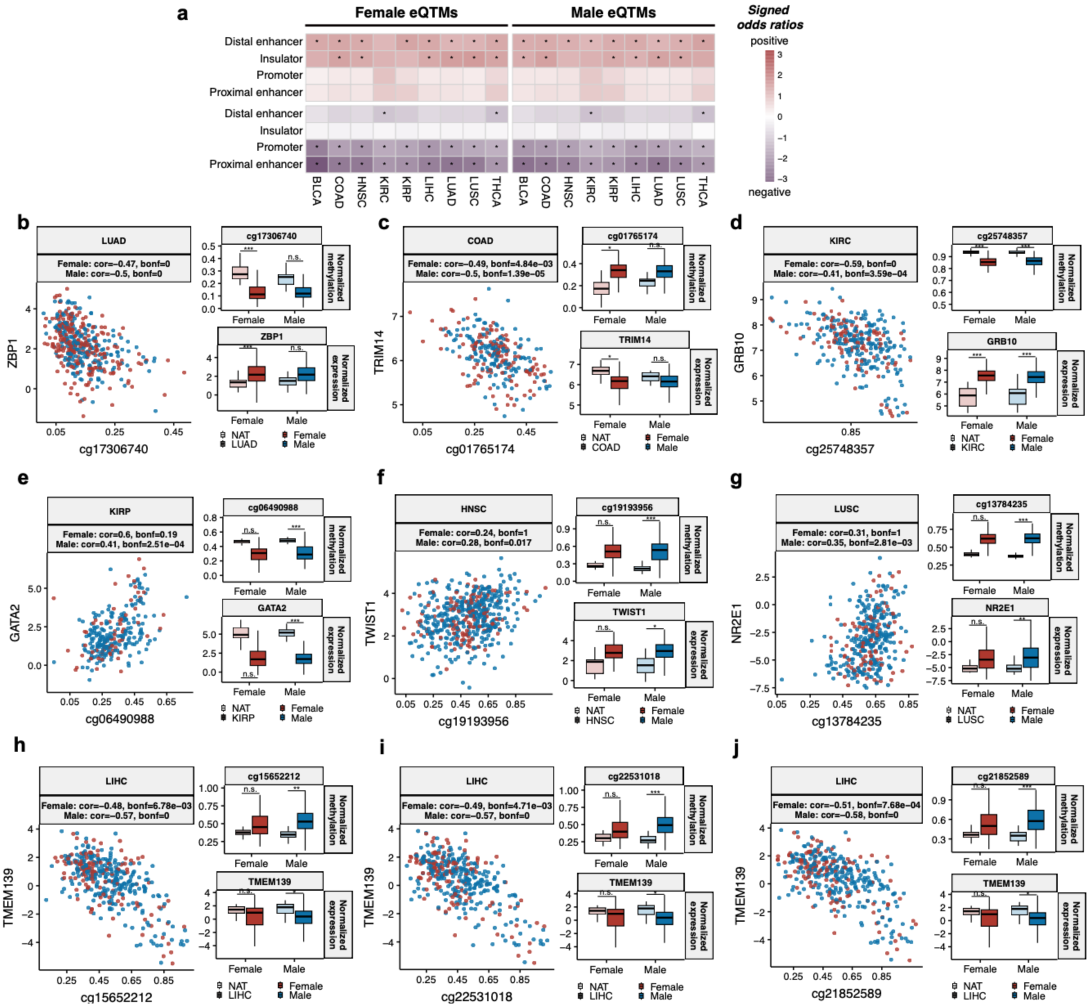

Supplementary Fig. 6 Characterizing the correlations between DNAm and gene expression. a, Enrichment of cCREs for eCpGs in negatively correlated eQTMs and positively correlated eQTMs, respectively. The signed odds ratios are labeled in colors (two-sided: negative and positive). Significant FDR corrected *P*-values are indicated (*FDR < 0.05). **b-d**, Examples of female-amplifiers that were significantly correlated with gene expression of target genes, exhibiting a consistently greater magnitude of gene expression changes in females compared to males. **e-j**, Examples of male-amplifiers that were significantly correlated with gene expression of target genes, exhibiting a consistently greater magnitude of gene expression changes in males compared to females. cCREs, candidate cis-regulatory elements; eCpGs, eQTM related CpGs.

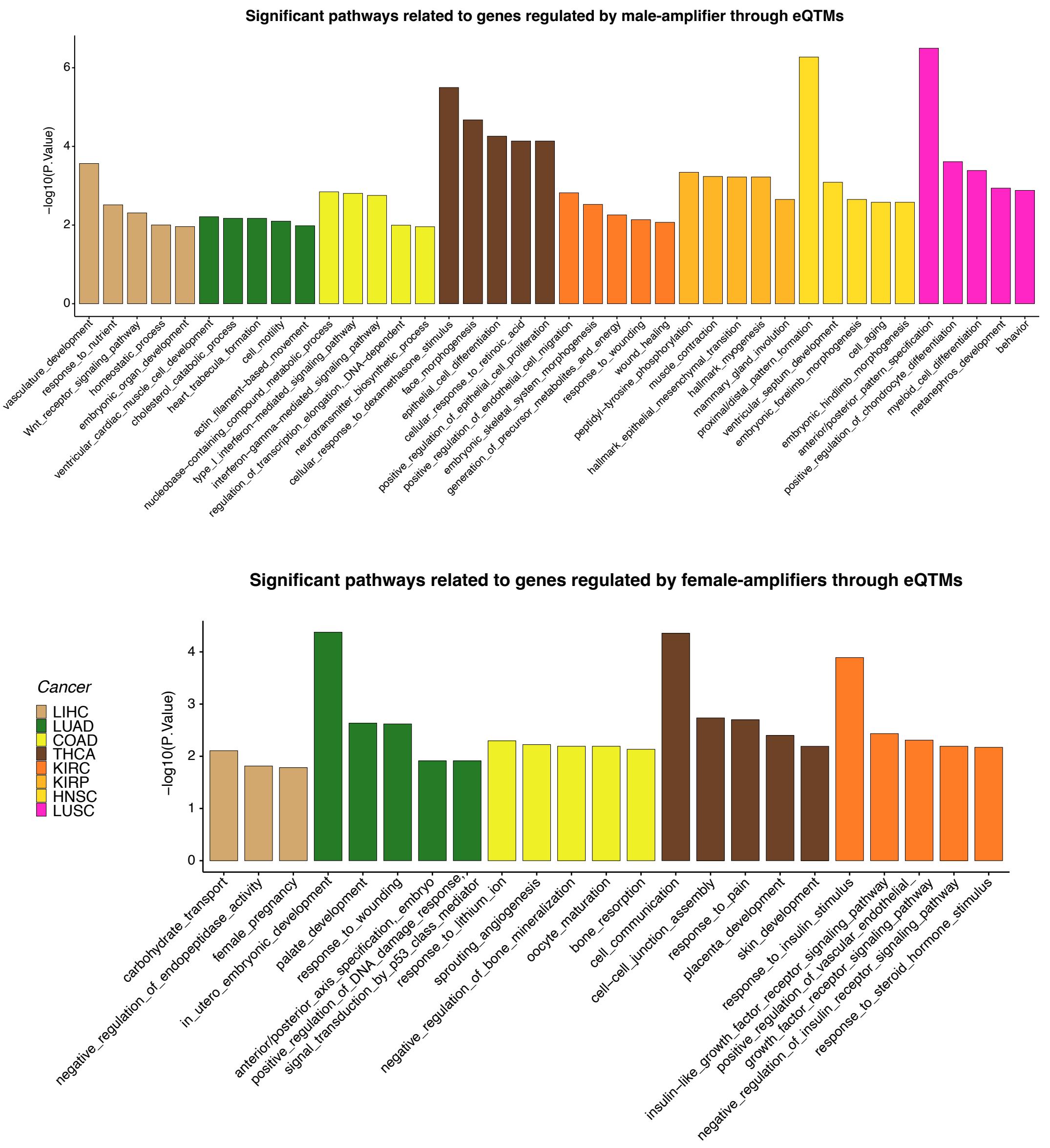

Supplementary Fig. 7 Functional enrichment for genes regulated by sex-amplifiers through eQTMs. Top five biological pathways for female- and male-amplifier related eQTM genes (*P* < 0.05 as the significant threshold).
